# The experiences of high school students with pulmonary tuberculosis in China: a qualitative study

**DOI:** 10.1186/s12879-016-2077-y

**Published:** 2016-12-15

**Authors:** Shaoru Zhang, Xiaohong Li, Tianhua Zhang, Yahui Fan, Yuelu Li

**Affiliations:** 1 Department of Nursing, Xi’an Jiaotong University, Xian, 710061 China; 2 Department of Nursing, Xi’an Medical University, Xian, 710021 China; 3 Shanxi Provincial Institute for TB Control and Prevetion, Xian, 710048 China

## Abstract

**Background:**

Clustered tuberculosis (TB) still occurred nationally in Chinese schools every year, where high school students patients accounts for the highest proportion. These young TB patients are in a critical period of physical and psychological growth. Research on their illness experience and analysis of underlying causes remains blank. The purpose of this study is to explore the overall illness experience of Chinese high school TB patients and to investigate the individual and social causes of such experience.

**Methods:**

Twenty-two high school TB patients in a certain county of Shaanxi province were interviewed in-depth twice when initial diagnosed and during intermediate treatment periods. Interview data were analyzed by framework approach.

**Results:**

The high school TB patients worried about interruption of studies rather than the disease. They generally showed a lack of awareness of tuberculosis, were highly dependent on parents, and received assistance from teachers and students during the treatment. Most of them did not show obvious stigma.

**Conclusion:**

The unique education system and sociocultural factors in China are the root of special illness experience of high school TB patients. Huge pressure in college entrance examination leads sick students to worry about interruption of studies more than the disease itself. Their serious lack of awareness of TB, caused by the ignorance of school, parents and the students, becomes the biggest obstacle to timely diagnosis and treatment. Whether high dependence on parents is conducive to disease recovery varies with each individual. Meanwhile, patients’ weak stigma could play a positive role in disease recovery. Educational and medical institutions should develop more effective TB control strategies based on these factors.

## Background

New cases of tuberculosis (TB) are decreasing gradually and its cure rate has slightly improved in China. However, China is still one of countries with high occurrence of TB, with about 1.3 million new cases annually, accounting for 11% of the global incidence and ranking second in the world [1]. According to the 2014 National Internet-based Infectious Disease Reporting System, 4.02% of TB patients is students. Twenty-one cases of clustered TB in schools had been reported nationwide from January 2009 to June 2013 according to the Management Information System for Public Health Emergencies. The average number of patients reported per case is twenty-five, of which fourteen are high school patients, accounting for 66.7%. High school students have become a high-risk group for clustered TB, incidence of which often substantially exceeds the average level of the local society once it outbreaks [2].

The Chinese government has long been focusing on the prevention and control of TB among student population [3]. In 1961, the Ministry of Education and the Ministry of Health jointly issued the *Notice on Prevention and Control of Infectious Diseases for Students: Tuberculosis and Hepatitis*, which was first to regard TB as a focus of infectious disease control in Chinese schools. In 2006, the ministries again jointly issued the *Specification for Epidemic Reporting of Infectious Diseases in School, Nurseries and Kindergartens (Trial)* to further strengthen the reporting of information related to public health emergencies such as outbreaks of TB and other infectious diseases in schools. In 2010, the ministries once again issued the *Specification for Prevention and Control of Tuberculosis in Schools (Trial)* to further strengthen the prevention and control of TB.

However, factors influencing infection, prevention and control of TB are complex and diverse. Poor nutritional status, smoking, poverty and lack of awareness about TB are considered the most important factors that increase the risk of exposure to TB [4–6]. Factors such as lack of knowledge about TB, patients’ perception of stigma not only affect the health seeking behavior of patients, but also contribute to long delay in diagnosis and poor adherence to TB treatment [7–12]. TB brings a lot of problems to patients and to their families. During the initial diagnosis period, TB patients often suffer anxiety, stress and other psychological reactions. Many TB patients experience negative emotions including irritability, tension, pessimism and depression due to illness stigma and social isolation during treatment [13, 14]. Factors like social situation, educational level, economic conditions and lifestyle of different patient groups have great impacts on the TB awareness and illness experience [15, 16].

Chinese high school students have long been under great pressure of study. They experience high stress, lack adequate sleep or effective exercise, have poor physical fitness and decreased immunity, which make them susceptible to TB infection. Meanwhile the students’ classrooms, dormitories and other environments are highly crowded, which also provide conditions for the outbreak of the epidemic [17, 18]. High school students are in a critical period of physical and psychological growth, which is a high-risk period for psychological conflict and behavioral, emotional problems as well [19]. They live in a relatively simple and closed environment, mainly study in schools and get care from their parents and other family members. They are under double supervision of school and parents and are behaviorally, economically dependent. After contracting TB, they will suspend schooling for treatment and recuperate at home in a semi-isolated state for about six months. The above factors make this group different from other social masses in thought and behavior.

Regarding researches on Chinese high school students as TB patient subjects, the most representative is W CHEN et al.’s report of a TB outbreak in China Shanxi’s Licai High School in 2011. They analyzed the causes and put forward feasible suggestions for TB prevention and treatment [2]. Successful cure should not only involve recovery of physical health, but should also concern the impact of the disease on patients’ mental health and life [20]. Better understanding of patients’ perceptions of TB and factors which influence that perception could help identify reasons for low or delayed accessing of health care, with recent WHO guidelines suggesting that ethical considerations and human rights issues should also be taken into account [21]. At present, research of overall illness experience targeting Chinese high school TB patients is still blank. The purpose of this study is to investigate in depth the illness perception and experience of Chinese high school TB patients and analyze its individual and social causes, in order to provide the basis for preventive treatment and for improving the life and well-being of these patients.

## Methods

### Study design

This study is a qualitative research, in which the illness experience and causes were investigated through in-depth interviews with high school TB patients. We adhered to CPREQ guidelines/methodology.

### Study setting

The research site was located in a certain county in north of Shaanxi province, China. It is a well-managed public high school, which is one of the local key schools, with part of students boarded and lodged at school. In March 2014, local high schools reported cases of TB, which caused high attention of the health departments. With the strong support of the local Center for Disease Control and Prevention (CDC), our research team conducted the first in-depth interview with patients at the local CDC and the county hospital in March to establish a mutual trust relationship and to understand the early-stage illness experience of patients. In July, infectivity was expected to have disappeared in most patients. Our team again visited the county CDC, and conducted a second in-depth interview with the same population to learn in detail about their illness experience during the intermediate treatment period.

### Participants

In this study, interviews were given to the same patient population at two time points: initial diagnosis period and intermediate treatment period. A total of twenty-two patients participated in the first interview, all of whom were high school students. They consisted of nine males and thirteen females, aging between 16 and 21 years. Two of the patients had a history of TB, while another patient had TB invasion to pleural cavity with abscess formation, whose condition was severe. A total of 18 patients participated in the second interview (four patients were lost to follow up due to withdrawal or visits to other hospitals). Among them, infectivity disappeared in 16 patients; one patient was hospitalized again due to recurrent disease; and the remaining one patient with a history of pulmonary TB discontinued medication in the fourth month of treatment due to poor efficacy. All subjects participated in this study voluntarily, who had normal language organization and presentation skills. General information of twenty-two sick students is shown in Table 1.

**Table 1:** General information of 22 high school patients with TB

	Gender		Grade			Origin		Whether received health education	
	Male	Female	G1	G2	G3	Rural	Urban	Yes	No
Number of cases	9	13	6	7	9	13	9	4	18
Percentage %	63.6	36.4	27.3	36.4	63.6	59.1	40.9	18.2	81.8

### Data collection

Interviews were completed by two interviewers. One of them is a professor of Xi’an Jiaotong University Medical School, who has long been engaged in the research of TB prevention and control. She once attended the qualitative research class held by Australian experts and conducted in-depth interviews with AIDS patients, college TB students, as well as school doctors and TB dispensary staff, and was highly experienced in qualitative research. Another interviewer was a lecturer of Xi’an Medical University, who was specializing in public health nursing. Two trained medical postgraduates served as the recorders of this study, who were responsible for text recording and audio recording interviews on site and for carefully observing the respondents’ non-verbal information.

With the support and cooperation of a Shaanxi’s certain county CDC, the research team visited the county twice in March and July of 2014 for on-site interviews and completed the collection of relevant information. In the first interview, sixteen of twenty-two subjects were interviewed in the single wards of the local county hospital’s infectious disease department, while the remaining six subjects were interviewed in the local CDC office. The second interview was conducted in the local CDC conference room. The interview environment was quiet and private. Each interview was about twenty-five to forty minutes. Before interviews, the interviewers described the purpose, methodology and confidentiality of the interviews and informed the necessity of text and audio recording the interview process. The participants understood the necessity of recording and the nondisclosure of their private information. Interviews began after signed informed consents of interviewees and their guardians were obtained. Interviews were carried out mainly around aspects such as illness and treatment experiences, impacts of illness on life and mental state, problems encountered in treatment, views on academic arrangement after illness and types of help expected (outlines of the two interviews are shown in Tables 2 and 3).

**Table 2:** Outline of first interview

1. How long have you been sick/receiving treatment? How did you find yourself sick? Please tell us your treatment-seeking processes?
2. What do you think might be the reasons for your sickness?
3. What are the problems and changes sickness has brought to you?
4. What is your biggest concern?
5. What kinds of help do you expect to get?

**Table 3:** Outline of second interview

1. How long have you been receiving treatment? Which examinations have you taken, and what are the examination results?
2. Did you strictly follow the doctor’s advices during treatment, or have you ever missed or discontinued any drug? What caused that?
3. Did you experience any side effect during medication? How did you overcome them?
4. What is your biggest concern at present?
5. Did you proactively access to TB-related knowledge during treatment? Through what channels?
6. What impacts have TB brought to you and your family? How do you see these impacts?

### Data analysis

In this study, the thematic framework approach was adopted. After completion of each interview, the audio records were transcribed to texts by researchers, who repeated listening and checking to perfect the transcripts and repeated reading of transcripts for encoding and classification to form themes. Before the panel discussion, researchers also conducted independent analysis, and inconsistencies were then repeatedly discussed by the panel to draw final conclusions.

## Results

### Disrupted daily life

After diagnosis, TB students immediately stopped schooling to begin a long treatment course, whose original order of life was disrupted.

#### Interrupted normal schooling

When asked about the biggest impact brought by the illness, all of twenty-two patients’ answer was discontinuation of schooling.

S6: I will soon take the college entrance examination, but now I can’t go to school and am unable to study in the ward as well.

S14: Now I can’t go to school, the school said that I can only go back to class two months later, which is quite a delay for study.

#### Limited self-care ability

The disease limited the patients’ self-care ability, with even the basic eating and dressing activities needing assistance of parents and family members. Some patients had severe conditions, where TB invaded into pleural cavity to cause abscesses and thus needed drainage and infusion therapies.

S3: Now everything has to be done by my parents. I can’t do anything by myself, even getting up from bed is not easy because of the bag (drainage tube).

S7: After I was hospitalized, many things are done with the help of my mom. She stays at the hospital all day to take care of me.

### Emotional reactions during treatment

During treatment, the patients experienced a wide range of emotions resulting from the illness.

#### Anxiety

Suspension of studies due to illness would undoubtedly affect the scores of unified examination and college entrance examination, so students were quite worried about this. In addition, the patients also feared that they might infect their family members and friends.

S8: The college entrance examination is approaching! Now it is already the second semester of grade two and soon it will be grade three. Anyway, there is not much time left (before the college entrance examination)

S10: I can’t continue my schooling now, because I fear that I might infect my family members and my classmates

#### Confusion

Many patients used to consider themselves physically healthy, so they were surprised and confused about their infection with TB.

S7: I consider myself having good immunity. Before the Spring Festival, a lot of my classmates caught a cold while I didn’t.

S13: I just didn’t expect to be infected with pulmonary TB. My health is so good that I thought I would be fine.

#### Guilt

Patients were deeply guilty for the living, psychological and economic impacts brought to their family members by their illness

S6: Well, I also worry that my family may be quite stressed. They think this disease a bit tricky.

S9: My family doesn’t make much money. Now I can’t study and my families are also worried. Plus, hospitalization is very troublesome, because they have to come to the hospital every day.

#### Gratification

During the suspension of schooling, patients were visited by classmates and teachers and received their care. They highly appreciated the help and support from teachers and classmates.

S5: Those who offered me help are classmates, teachers and family members. Some of my best friends gave me calls. Once I went to school, my teacher said a lot of words to encourage me in the class meeting.

S19: Many classmates came to visit me, both past and present. They all encouraged me not to worry about the study.

### Academic and health expectations

After receiving treatment for nearly five months, the majority of students were no longer infectious and restored to good health. Poor efficacy was found only in two students. When asked about the biggest concern at present, sick students expressed their concerns about studies and their own health.

#### Return to school

After receiving regular treatment, the disease was effectively controlled for the vast majority of sick students, along with fatigue disappearance. They desperately wanted to catch up the new semester and resume studies as soon as possible.

S3: School will open in September again and I’m eager to go to school. Now my health has recovered and I can no longer stay at home and delay my study.

S5: I once went to school with several classmates and we all wanted to return to school as fast as possible.

#### Academic catch up

Several sick students stated that they had excellent grades before contracting TB, whose studies were delayed now due to infection with TB. They were extremely worried about whether they can catch up and restore their original status.

S9: I originally attended the experimental class and now five months have passed since I first received treatment at home. I worry that I can’t keep up with the progress of classmates after returning to school and restore my original grades.

S22: The teacher told me that I have to drop one grade (repeat grade two) if I want to resume studies, because I certainly can’t keep up with the grade three curriculum. But I also fear that if I repeat grade two but have poor grades in the new class, it will be very shameful.

#### Health recovery

students with severe TB conditions were still receiving active treatment, whose major concern was how to fight the disease and restore health as soon as possible.

S10: Examination results are not good for these several times. I’m also adequately cooperating with the treatment, hoping to get well soon.

S13: The doctor said that this disease can’t be rushed. My condition has not recovered fully and I’m currently not thinking about school. My parents also want me to recuperate first.

### Ideological problems about treatment and recovery

High school students did not really understand the seriousness of TB due to lack of sufficient awareness, which was not conductive to treatment and recovery.

#### Unclear about the cause

The sick students could only guess the causes of TB to be catching a cold or infection by others, who were unclear about the real causes.

S9: We had an activity outside then and ate outside. It might be due to eating something (bad) outside.

S20: I don’t know (the reason). I was always in good health. When I felt uncomfortable those days I went to the hospital and had a bad fever after several days.

#### Lack of knowledge about TB

Despite undergoing several months of treatment, the sick students’ understanding of TB knowledge was still unsatisfactory. They have not grasped basic knowledge such as symptom differentiation, transmission routes and preventive measures yet.

S1: I know that I will get infected if others use my tableware during dining.

S15: I didn’t have any symptoms when I caught TB. I think knowing those symptoms is of no use.

#### Lack of initiative to access to information

When asked about whether they have ever accessed to the TB-related information, only a small number of them said that they have consulted information initiatively, while most students stated that they have little interest in this regard.

S2: I occasionally see TB-related contents when surfing the Internet. I rarely look them up intently.

S4: I’ve seen TB-related leaflets being delivered on the streets. I thought it (TB) was a minor illness and so didn’t want to know about it.

#### Weak awareness of standard treatment

Interviews found that one sick student with a history of pulmonary TB discontinued medication voluntarily due to poor efficacy. Besides, a small number of students occasionally missed medication.

S4: I usually remember to take medicine, but occasionally forget to take them and make it up once I remembered.

S10: I had caught this disease (TB) before and this time, medication was still ineffective. Plus, I also spent additional money for buying liver-protective drugs. So I discontinued the medication.

## Discussion

This study is the first to investigate in depth the illness experience of Chinese high school TB patients. We found that patients were worried most about interruption of studies rather than the disease itself. They were generally lack of awareness of TB, were strongly dependent on parents, and received support and assistance from teachers and students. However, different from past research results on other groups, the majority of high school patients did not feel obvious stigma.

### Interruption of studies was the primary concern

Patients were worried most about studies and examinations instead of the disease itself. After being diagnosed with TB, patients stopped schooling immediately for treatment, whose normal studies were interrupted. Although China’s guidelines for TB prevention and control state that the infectivity disappears in those infected with pulmonary TB after two to three weeks of regular treatment, who can participate in social activities, many schools require the patients to return to school after complete cure (six to eight months) in order to prevent the spread of epidemics. Long-term suspension of schooling often leads to decline in grades, and in the case of higher grade students, they may even unable to take the unified examination or the college entrance examination. We believe that such priority to studies and examinations over health stems from China’s college entrance examination system.

In China, it is related to each student’s future to be admitted to a good university and become the “chosen one”, especially for the majority of students from common families because this means change of fate and improvement of social status [22, 23]. China has a large population base, with the number of annual college entrance examination applicants exceeding 9 million or even 10 million in recent ten years. However, high-quality higher education resources are extremely scarce. National average admission rate for key universities (first line universities) is only 8% and for “Project 211” universities even only 5%. The public describes the college entrance examination as a stampede of “thousands of soldiers and horses crossing a single-plank bridge”; the competition is fierce. China’s current college entrance examination system does not take quality assessment, classroom performance, usual grades or recommendation letter as admission references, where the situation is basically “one examination decides whole life”. If a student fails examination, he or she can only take it again in the next year as a reviewing schooling student, which greatly increases time cost, psychological pressure and family’s financial burden. Therefore, students study from dawn to night, making use of every possible minute. Even in many regions, study in schools takes more than 16 h every day and the students rest for a day only once every two to four weeks. Time is extremely valuable to them.

Up to six months of absence leads the patients to severely fall behind the syllabus schedule. Even if they return to school after recovery, the long-term absence will cause decline in learning state, which will result in their lack of self-confidence and reduced learning ability. Especially in the final sprint stage of the third year, suspension of schooling will be a heavy blow to students. Many students stated that they were helpless against such suspension arrangements. They hoped to be able to study during treatment and get help and support on studies, especially for the high grade patients.

### Lack of awareness was the biggest obstacle to diagnosis and treatment

In the early stage, TB manifests itself by cough, fever and other symptoms similar to common cold or fever, during which patients generally do not know they have caught TB. In particular, patients who think themselves in good health will not consider the possibility of TB at all and relax their vigilance. They are unclear about the real causes of disease. Many students do not seek immediate medical attention, and some even drag on for several weeks to go to the hospital. This not only delays treatment and aggravates the condition, but is also prone to outbreak of clustered TB as the patients maintain everyday life and contact with people [24]. After diagnosis, almost all patients consider TB not a serious disease, which is rather easy to cure. They are lack of understanding of the longevity and complexity of treatment from recovery, have weak awareness of standard treatment, and almost do not initiatively access to TB-related knowledge [25]. This differs enormously from other infected populations in terms of awareness of the condition and urgent need for TB-related knowledge [26]. This not only stems from the students’ own problems, but also reflects the schools’ and families’ insufficient efforts in health education.

On the one hand, Chinese high school students focus on how to improve test scores, who often do not have the energy, time and willingness to understand the knowledge of TB; they think TB infection is highly unlikely to happen to them and do not pay enough attention to the health education organized by their schools. After contracting TB, they are informed that TB can be cured and is basically not life-threatening, so they do not worry too much about the condition and are even blindly optimistic, who relax vigilance on the longevity and complexity of treatment. Moreover, the vast majority of high school students will not immediately face employment, marriage and other significant life events, who have no conception of the possible adverse effect of previous history of TB [27]. As the saying goes, “ignorance makes fearless”. Lack of overall awareness of TB has resulted in the patients’ insufficient attention to TB and unwillingness to explore relevant knowledge initiatively.

On the other hand, TB prevention and treatment education in schools and families is very weak as well. Although the *Specification on Prevention and Control of TB in Schools (Trial)* includes the TB prevention and treatment knowledge as an important part of health education, Chinese schools tend to take college admission rate as the absolute focus of work, neglecting health education on infectious diseases. Thus health courses are characterized by short duration, mere formality and lack of faculty, which have little educational effect. Meanwhile, most parents are lack of knowledge of TB themselves, who therefore are unable to educate their children [28]. In our present study, eighteen of all twenty-two patients have not received any health education, accounting for 81.8%. Students can only get scraps of knowledge about TB from media such as television, radio and Internet, whose grasp of knowledge about TB prevention and control is far away from the requirements of the *National Tuberculosis Control Program 2011–2015* [29]. Publicity and education on TB control should be urgently strengthened.

### Reliance on parents was a common tendency during treatment

In the interviews, patients indicated that during hospitalization and recuperation at home, their parents and family members assumed full responsibility for taking care of their diet and daily life, and some even did not hesitate to suspend work. Without their care, patients did not know how to cope with the disease, reflecting the high degree of dependence on the family. We believe that this is closely related to the social factors and educational ideas in China.

Firstly, Chinese families ubiquitously hold high hopes for their children [30]. In the fiercely competitive modern society, the traditional ideas of “a good scholar will make an official” and “Intellectuals are the most superior” become highly popular, while good grades and admission to a good university are the first step to success. Therefore, parents are obsessed with their children’s improvement of academic performance and willing to take care of anything else no matter how hard it is. High school students rarely initiatively undertake affairs unrelated to studies due to the study pressure. They generally form a habit of relying on family members. Schools, on the other hand, emphasize college admission rate, while concern little about improving students’ self-care ability and even encourage parents to take care of their children’s daily life so as to save more study time for them. Secondly, although patients have not lost their self-care ability during recuperation, parents even handle everything in order to let their children recover more quickly, thereby prompting the patients to relax self-demands and throwing the responsibility of daily life and treatment recovery to parents. In addition, patients are psychologically not mature enough, who cannot bear the stress caused by recurrent illness and mood swings alone. Enhanced role as patients also drive them psychologically more dependent on their parents. Thus, patients were more strongly dependent on parents in terms of both daily life and mental state. A major advance in improving TB treatment compliance is the implementation of DOTs program, which requires every TB patient to have a partner for monitoring their daily medication [31, 32]. If parents can provide patients with strong daily care and spiritual support, it will be enough to compensate for the lack of their own responsibility for treatment. Such strong dependence may be conducive to patients’ recovery; otherwise, it may cause their further neglect of concern over their own condition, which will result in poor recovery.

### Weak stigma was a favorable factor in diagnosis and treatment

Many studies on TB have described the ubiquitous issues of stigma and discrimination [33–35]. However, in the present interviews, the patients did not show obvious stigma, who were actually quite at ease during the interviews. According to the research of Sushil C Baral et al. [8], stigma of TB patients generally comes from self-discrimination and public discrimination. Reasons for self-discrimination lie in worry about spread of TB, avoidance of gossip and potential discrimination from others. Reasons for public discrimination include fear of being infected, association of TB with poverty and vulgar, association of TB with alcoholism, smoking, prostitution and other misconducts, belief that TB is God’s punishment, etc. However, as Chinese high school TB patients and their surroundings might have no condition or motive to bring the above discriminations, they did not show obvious stigma.

Firstly, as mentioned above, the patients did not realize the seriousness of the disease. They thought that they would soon be cured and resume normal life, and did not feel inferiority due to illness. Secondly, they were generally well isolated and would not easily infect others, so they did not have the psychological burdens such as guilt. More importantly, as the present case was a clustered outbreak, patients would not attribute the illness to their own reasons. Boarding life and home-school state make the public not link the illness to the individual behaviors. Besides, they received comprehensive care from parents and support from teachers and classmates during the hospitalization and recuperation at home. They were not deprived of social support because of illness or external discrimination. Therefore, patients did not have obvious stigma; meanwhile, external social support can play a very positive role in the recovery of the condition [36, 37].

## Conclusion

This study explores in depth the illness experience of Chinese high school TB patients and its sociocultural roots, which is of considerable value in providing them with psychological and social supports. China’s unique education system and sociocultural factors are the root causes of special illness experience of high school TB patients. Huge pressure in college entrance examination leads sick students to worry about interruption of studies more than the disease itself. Their serious lack of awareness of the disease, caused by the ignorance of school, parents and the students, becomes the biggest obstacle to timely diagnosis and treatment. Whether high dependence on parents is conducive to disease recovery varies by sick student. Meanwhile, patients’ weak stigma can play a positive role in disease recovery. Educational and medical institutions should develop more effective TB control strategies based on these factors.

During the interview process, we also conducted health education seminars and answered patients’ questions, benefiting nearly ten thousand students and teachers.

### Limitations

This study adopts a convenience sampling approach, where only sick students treated in the local hospital are selected as subjects. Due to operational difficulties, some patients who were not hospitalized timely after diagnosis or treated outside the province are not included as the interview subjects. Illness experience of interviewees also varies due to different treatment time and efficacy, which may have some influence on the analysis of research results.

## Abbreviations

CDC: Center for disease control and prevention
TB: Tuberculosis
